# Enabling public, patient and practitioner involvement in co-designing frailty pathways in the acute care setting

**DOI:** 10.1186/s12913-019-4626-8

**Published:** 2019-11-05

**Authors:** Deirdre O’Donnell, Éidín Ní Shé, Mary McCarthy, Shirley Thornton, Thelma Doran, Freda Smith, Barry O’Brien, Jim Milton, Bibiana Savin, Anne Donnellan, Eugene Callan, Eilish McAuliffe, Simone Gray, Therese Carey, Nicola Boyle, Michelle O’Brien, Andrew Patton, Jade Bailey, Diarmuid O’Shea, Therese Cooney Marie

**Affiliations:** 1 School of Nursing, Midwifery and Health Systems, University College Dublin, Dublin, Ireland; 2 Expert by Experience Representing the Older People’s Empowerment Network, Dublin, Ireland; 3 Expert by Experience Representing Family Carer’s Ireland, Dublin, Ireland; 4 Expert by Experience Representing Sage Advocacy, Dublin, Ireland; 5 Sage Advocacy, Dublin, Ireland; 6 Expert by Experience Representing Glór and Age Action Ireland, Dublin, Ireland; 7 Expert by Experience Representing the Disability Federation of Ireland, Dublin, Ireland; 8 St. Vincent’s University Hospital, Elm Park, Dublin, Ireland; 9 School of Medicine, University College Dublin, Dublin, Ireland

**Keywords:** Co-design, Public and patient involvement, Frailty, Older people, Health system, Person-centred care

## Abstract

**Background:**

Although not an inevitable part of ageing, frailty is an increasingly common condition in older people. Frail older patients are particularly vulnerable to the adverse effects of hospitalisation, including deconditioning, immobility and loss of independence (Chong et al, J Am Med Dir Assoc 18:638.e7–638.e11, 2017). The ‘Systematic Approach to improving care for Frail older patients’ (SAFE) study co-designed, with public and patient representatives, quality improvement initiatives aimed at enhancing the delivery of care to frail older patients within an acute hospital setting. This paper describes quality improvement initiatives which resulted from a co-design process aiming to improve service delivery in the acute setting for frail older people. These improvement initiatives were aligned to five priority areas identified by patients and public representatives.

**Methods:**

The co-design work was supported by four pillars of effective and meaningful public and patient representative (PPR) involvement in health research (Bombard et al, Implement Sci 13:98, 2018; Black et al, J Health Serv Res Policy 23:158–67, 2018). These pillars were: research environment and receptive contexts; expectations and role clarity; support for participation and inclusive representation and; commitment to the value of co-learning involving institutional leadership.

**Results:**

Five priority areas were identified by the co-design team for targeted quality improvement initiatives: Collaboration along the integrated care continuum; continence care; improved mobility; access to food and hydration and improved patient information. These priority areas and the responding quality improvement initiatives are discussed in relation to patient-centred outcomes for enhanced care delivery for frail older people in an acute hospital setting.

**Conclusions:**

The co-design approach to quality improvement places patient-centred outcomes such as dignity, identity, respectful communication as well as independence as key drivers for implementation. Enhanced inter-personal communication was consistently emphasised by the co-design team and much of the quality improvement initiatives target more effective, respectful and clear communication between healthcare personnel and patients. Measurement and evaluation of these patient-centred outcomes, while challenging, should be prioritised in the implementation of quality improvement initiatives. Adequate resourcing and administrative commitment pose the greatest challenges to the sustainability of the interventions developed along the SAFE pathways. The inclusion of organisational leadership in the co-design and implementation teams is a critical factor in the success of interventions targeting service delivery and quality improvement.

## Background

Frailty considers the complex interplay of physical, psychological, social, and environmental factors and is associated with key clinical syndromes including loss of mobility, falls, confusion, incontinence and polypharmacy [1, 2]. Although not an inevitable part of ageing, frailty is an increasingly common condition in older people. Frailty affects approximately 8–10% of people over the age of 65 and 25–50% of those aged 85 and over [3–5]. Frail older patients are particularly vulnerable to the adverse effects of hospitalisation, including deconditioning, immobility, loss of independence [6, 7]. Recent attention has focused on identifying the best pathways for treating frail older patients. Studies illustrate the importance of continuing organisational support, clinical champions who communicate regularly with decision makers, dedicated staffing, and ongoing data collection [8, 9].

In Ireland, as in other jurisdictions, people are living longer and can expect to live without disability or morbidity for longer than ever before. Over the next 30 years, the over 80 population is set to rise dramatically; increasing from 128,000 in 2011 to 484,000 by 2046, under positive migration assumptions [10]. While this is a welcome and positive outcome from advances in health and social care it heralds a policy crisis in relation to planning and preparing for demographic change. Demand for health and social care, related to an ageing population, is projected to increase across all sectors in the years to 2030 [10, 11]. This generates concern about whether policy and service planning is in place which will enable the health system to respond to meet this demand [11]. A recent review of the data from the Irish longitudinal study on ageing found that frailty was a significant predictor of utilisation of most social care and medical care services [12]. Improving care for frail people is a national priority [11] and an important aspect of the National Clinical Programme for Older People in Ireland. However, very little evidence is available regarding implementation of strategies in the Irish context to improve care for frail older people [13].

Our focus within the ‘Systematic Approach to improving care for Frail older patients’ (SAFE) study was to co-design quality improvement initiatives within an integrated care pathway for frail older people in an acute setting [2]. As part of the SAFE study we undertook a review of the literature to capture what works in the successful implementation of a frail older person’s pathway [14]. A significant finding in the review was the absence of methodological studies which include patients and public groups in the co-design of frailty interventions and in the identification of priority areas for improvement initiatives. The review identified two grey literature reports that stressed older people are experts in their own care through their lived experiences and they must be included and consulted in the design of any frailty pathways [15, 16].

Key to enabling the involvement of public and patient representatives (PPRs) in the design of frailty pathways is the utilisation of co-design methodologies and approaches that empower and include the perspectives of older people and their family carers [12, 17–19]. The literature highlights many challenges in enabling meaningful reciprocal engagement [14, 15, 17–19]. A co-design approach within health system improvement initiatives involves creating an equal partnership of people working within the system and those individuals who have lived experience of using the system (patients and their families/carers) [2, 14, 20–22]. Co-design in particular requires all participants to move out of their ‘comfort zone’ and be willing to engage with a diverse group [16, 23].

This paper presents a systematic approach to involving public and patient representatives (PPRs) in the co-design of care pathways for acute frail older patients [2, 24]. Through the meaningful engagement of PPRs five priority areas were identified for targeted quality improvement in the care of frail older people in an acute hospital setting. This paper outlines how these priority areas were embedded into the pathway enhancement through the identification of quality improvement initiatives aligned to outcomes which are patient-centred and meaningful for all involved in the process.

## Methods

A co-design team was established at the commencement of the SAFE study to design and develop the frailty pathways for older people. The team was composed of older people, carers, organisations advocating for older people, academic researchers as well as clinical personnel from the acute hospital site as well as the integrated community care team (ICT) for older people. The co-design approach adopted in this project was guided by principles of authentic participation and collaboration [20].

### Co-design participant recruitment

Ten participants were recruited from the membership of NGO and community-based patient and public advocacy organisations. The public/patient participants included older people with recent experience of acute care (*N* = 6), advocates for frail older people in hospital (*N* = 2), a family carer of older people with recent hospital admissions (*N* = 1) as well as a frail person living with a degenerative disability necessitating a wheelchair and who had frequent hospital admissions (*N* = 1). Eight healthcare practitioners were recruited to the team. Included were geriatric and acute care physicians that included that National Clinical Lead for the Care of Older People (O’Shea) as well as a clinical nurse specialist for older people’s care. Three academic researchers (including a research assistant) were also participants. The eight practitioners were involved on a rotating basis ensuring a critical mass of public/patient participants. There was consistently greater representation from public/patients than the professional (average = 5 per meeting with a maximum of 8) or researcher participants (*n* = 3).

Six co-design meetings were facilitated over an 18 month project period. These were structured consensus building workshops intended to ensure the resulting integrated care pathway for frail older people was patient-centred and responsive to identified and prioritised service delivery need. With the consent of the participants, the meetings were audio recorded for transcription and detailed notes of each meeting were undertaken by a researcher (ÉNS). The researcher recorded the main discussion points and presented discussion summaries to participants for validation after each meeting. The first four co-design meetings ran in parallel to the pathway development. Thereby intervention implementation was conducted alongside the co-design process ensuring user responsiveness and public/patient driven iterative adaptation (Appendix 1).

### Methodological approach for meaningful co-design

A co-design methodological approach facilitates democratic dialogue in the development and implementation of change interventions and service improvement [22]. The approach adopted for this study facilitated co-design alongside service improvement implementation and evaluation. In this way the service design, implementation and evaluation were occurring in tandem and through iterative cycles of Plan, Do, Study and Act [25]. There is an inherent fluidity between process and outcome which characterises a co-design approach to the implementation of improvement initiatives [26, 27]. In this way, the process of co-design both informs and is informed by outcome identification and evaluation.

The methodological approach adopted for this co-design work was supported by four pillars of effective and meaningful public and patient representative (PPR) involvement [26, 28]. These pillars were: research environment and receptive contexts; expectations and role clarity; support for participation and inclusive representation; commitment to the value of co-learning involving institutional leadership.

#### Research environment and receptive contexts

The importance of fostering a positive team atmosphere which is receptive to the contribution of PPRs was noted as being a critical factor enabling meaningful engagement [28]. The strategies adopted in order to establish an environment conducive to co-design included the use of democratic dialogue and external facilitation. The academic participants in the SAFE co-design meetings co-chaired the co-design meetings and focused on enhancing PPR input into the process. These academics were experienced in the conduct and management of group discussions and in ensuring the equal and democratic representation of all voices at the table. In particular, one of the PPR members acted as a co-chair of each meeting alongside the academic member (McCarthy and Ní Shé). Furthermore, the representation of the clinical staff was rotated ensuring a higher proportion of PPRs at each meeting thereby amplifying patient voice and input [26].

The PPRs were offered capacity building sessions prior to engagement in the co-design process in terms of education regarding the project objectives, familiarity with the context of integrated care policy and service delivery for older people as well as an introduction to the organisational leaders who were engaged with the project. The academic co-chair was a point of contact for all PPRs and often held informal conversations outside of meetings to capture additional insights which were recorded in field observations. The meetings were conducted in the same accessible location with lunch and refreshments provided at each session. The facilitators aimed at developing a relaxed atmosphere with informal language, the prohibition of jargon and explanation of technical language and the various roles of health service personnel involved in the delivery of care for older patients.

#### Expectations and role clarity

Realistic, equitable and well-articulated expectations regarding participant input into the co-design process were highlighted as important enablers for meaningful engagement [28]. The PPR participants were nominated by advocacy and NGO organisations to which they were members and invited to participate in this project in order to share their valued and valuable experiences of acute care of older people. Terms of reference were agreed at the first co-design meeting in which participant roles were clarified and defined. The overall goal of the project was identified as being to develop integrated care pathways for frail older people which would enhance care processes and service delivery. It was agreed that the PPR role was to reflect upon and share their personal experiences of health service delivery to older people. The PPR members agreed to assist with the prioritisation of issues of concern and to assist with the identification of outcome measures to assess performance and enhanced patient outcomes. It was agreed that the role of clinical staff co-design participants was to provide contextual insight into health service delivery for older people in the context of the acute hospital site. Furthermore, the clinical staff were responsible for implementing the prioritised action points identified by the co-design team and reporting back to the team as to implementation performance. The role of academic staff participants were to facilitate the co-design meetings, translate the prioritised goals from each meeting and assist with the reporting and feedback regarding performance as well as dissemination of project outputs.

#### Support for participation and inclusive representation

Financial and administrative supports as well as wide public/patient representation during the research process have been noted as critical enablers of effective and meaningful PPR engagement in research and co-design of service delivery [16, 26, 28]. This project aimed for an inclusive and wide representation of patients and public collaborators. Recruitment of co-design members was channelled through advocacy and NGO organisations representing older people and those with disabilities in the Irish health and social policy context. Older people with experience of acute hospital care were the primary target for participant recruitment. A family carer of an older person who had experience of acute hospital admission was also included as it was agreed that family carers are an important stakeholder in the design of service delivery for frail older people. Furthermore, a participant with complex needs related to disability was included in the group as it was agreed that he provided critical insight into issues relating to universal design, accessibility and how service delivery structures can disempower individuals with disabilities associated with frailty. Financial compensation for any costs associated with participation (travel, subsistence, care release) were met by the project team funded by the study. A research assistant for the project acted as a coordinator; providing documentation and scheduling meetings etc.

#### Commitment to the value of co-learning involving institutional leadership

Securing institutional commitment, sponsorship and leadership for the co-design process has been identified as a key mechanism for fostering meaningful and valuable patient/public engagement in service delivery and design [28]. The SAFE study received funding from a national applied research scheme which ensured a partnership approach between a research lead (Cooney) and a knowledge lead (O’Shea). The purpose of this partnership was to ensure research findings were applied by the knowledge user organisations represented by a knowledge lead with the capacity and authority to implement research findings into policy and practice [2]. This project benefited from the involvement and commitment of the National Clinical Lead for the Care of Older People (O’Shea) who attended co-design meetings on a rotating basis. Furthermore, consultant leads at the local hospital site were engaged as were nurse management and department heads relating to allied health professionals. This was a critical factor ensuring that the PPR participants understood that their co-design contributions were being valued and it also guaranteed institutional support for the implementation of the co-designed pathway.

## Results

In response to the co-design team input a network of integrated care pathways for frail older people was envisaged. The co-design work prompted service and quality improvement initiatives along these care pathways beginning with frailty screening in the Emergency Department [29]. Each of the improvement initiatives directly corresponded to patient-centred outcomes identified by the co-design team and were aligned to five priority areas (Appendix 2). The five priority areas were: collaboration along an integrated care continuum for older people, continence care, improved mobility, access to food and hydration and improved information and signage. As phased implementation of the pathways is ongoing, the service and quality improvement initiatives which were developed in response to the co-design work, are being evaluated using a Plan Do Study Act (PDSA) process [25]. This will generate an iterative process of intervention development whereby the co-designed models of care are continually adapted to the context of the Irish health system and the specific acute hospital site (Table 1).

**Table 1:** Five priority areas for enhanced service delivery; related interventions and patient-centred evaluation outcomes

Priority Area	Interventions	Co-design Outcomes
Collaboration along an integrated care continuum for frail older patients	a) Early identification of frail patients upon admission b) Addressing organisational barriers on integrated care pathway	Rockwood frailty: Numbers screened Development of frailty index and its association with length of stay, mortality and discharge destination Improved bi-directional flow between primary care, acute and community based rehab or step-down institutions Improved discharge planning processes to the integrated community care team Rapid access pathways between GP and day hospital (bi-passing ED).
Continence care	a) Intentional rounding (IR) b) HCA skills fare	Personal needs Access to call bell and drink Clutter free bed space Access to sensory equipment Number of falls
Improved mobility	a) Introduction of FITT team in the emergency department b) End PJ Paralysis scheme	Hours from ED admission to first FITT therapy attendance (OT, PT, Dietetics and SLT) Numbers screened as frail who had FITT service and their average length of stay Patients mobilising on the ward Patients sitting out of bed on the ward
Access to food and hydration	a) HCA dedicated role in ED b) Intentional rounding c) Red Tray d) HCA skills fare	Access to a drink on ward (IR) Access to a drink in ED (HCA) Energy and protein consumption
Improved patient information and signage	a) IR and use of notice boards on ward b) Written daily care plans with goals c) Patient information leaflet regarding mobilisation d) Establishment of Environmental Dementia Committee	Comment and feedback from patients regarding information dissemination Signs at the correct height Writing large enough and easy to read (Colours and readability) Patients able to find their way around using signs alone

### Collaboration along the integrated care continuum

Inter-agency collaboration was consistently identified in the co-design discussions as a critical enabling factor for enhancing integrated care processes and health service delivery for frail older people. The co-design participants spoke about the lack of information following the patient through their care journey. Information sharing was seen to be critical to inter-professional collaboration as well as avoiding repeated assessments within the same acute admission. This collaboration incorporated the identification of frail older people at the point of admission to the emergency department in order to allocate the appropriate targeted care pathway within the hospital. Furthermore, the participants spoke about the necessity to connect services through the patient journey from the community to the acute setting and back to the community.

During the co-design discussions, it was noted that good inter-professional collaboration is contingent upon a high functioning cross agency case management approach with clearly identified roles and collective responsibility for effective care delivery. This understanding of the case management approach necessitated the addressing of organisational and disciplinary boundaries along the integrated care pathway. The nexus points of hospital referral and admission as well as discharge were identified as being important targets for improved collaboration along the integrated care continuum.

The interventions prioritised by the co-design team which would target the theme of ‘collaboration’ included: the introduction of a frailty screening tool at emergency department triage which facilitated identification of patients for cross-disciplinary frailty interventions [29]; the development of a frailty index enhancing a common understanding of associated risk factors for frailty; the development of direct referral pathways for primary care general practitioners to a Rapid Access Treatment day hospital site; implementation of a bi-directional flow between the acute setting and discharge destinations including rehabilitation sites, day hospitals and the integrated community care team. Much of the interventions co-designed under this prioritised theme involve inter-personal and cross disciplinary relationship building, the fostering of trust across organisations and the enabling of professionals and organisations outside of the acute setting in relation to referrals and delivery of targeted health services.

### Continence care

Improved continence care was identified as a priority area for intervention for older people admitted into the Acute Medical Unit and/or the geriatric ward. The PPR members of the co-design team emphasised this aspect of care as being of central importance to their understanding of quality person-centred care particularly as it pertained to dignity and respect for the individual patient.

In order to address the priority area of continence care for frail older patients the co-design team agreed to pilot test a paperless Intentional Rounding (IR) intervention in a busy 27 bed medical ward specialising in endocrine and renal specialties and which included frail older patients [30]. IR is a structured approach to patient care through conducting scheduled rounds [31]. Incorporated in the version of IR developed for the SAFE project was an opportunity to ask each patient if they required help with any aspect of continence. This was made a central aspect of care rounds in order to support patients to maintain continence while they were in hospital and prevent the loss of function which is associated with hospitalisation in frail patients.

A complete description of intervention development, implementation and evaluation is presented by Gray et al. [30]. The intervention involves the development of an IR protocol which includes the identification and management of patient’s personal needs including continence care. As part of the PDSA evaluation baseline and post intervention measurements of care are obtained through clinical audit and compared in order to identify improvements associated with the IR intervention [30]. This intervention should be complimented by a skills fare targeting healthcare assistants in the hospital (HCAs) which include a component on continence care for older patients. Following the success of the intervention in the pilot site and with the support of nursing practice development, IR is being embedded into nursing practice across the hospital.

### Improved mobility

During the first and second co-design workshop it was noted that frail older people are particularly vulnerable to deconditioning, immobility and loss of independence as a consequence of hospital admission. The PPR participants considered whether a mentality which associated hospital care with bed rest may be compounding the risk for detrimental outcomes following a period of admission. The PPRs emphasised the emotional and psychological impact of staying in bed and associated being up and dressed in their own clothes as a powerful indicator of patient dignity and empowerment. Interestingly, the conversation turned to patients’ choice of clothes and how this related to individual sense of identity. The clinical participants at the meeting suggested advising patients to bring comfortable clothes for example running shoes and leisure trousers. The PPR members noted that people should be advised to bring the clothes that they would normally wear at home and which they felt comfortable with in terms of their personal sense of self.

In order to improve patient mobility it was agreed that two initiatives should be introduced into the acute site. The Frailty Intervention Therapy Team (FITT) involves the identification of patients screened as frail to be reviewed by a multidisciplinary team for the early intervention while in the ED. The early intervention therapies represented by the team include occupational therapy, physiotherapy, nutrition and dietetics, speech and language therapy, pharmacy and social work. The FITT intervention was successfully piloted in the acute hospital site for two 3 month periods (November 2017–February 2018 and December 2018–February 2019) and subjected to PDSA cycles of evaluation demonstrating: reduction in time from admission to first therapy attendance; reduction in overall length of stay; as well as a reduction in readmission rates.

An initiative to encourage patients to get out of bed and get dressed was introduced as part of the Intentional Rounding (IR) intervention and a pilot End PJ Paralysis intervention was developed for implementation into a 22 bed care of the older person ward. A patient information leaflet was developed by the co-design team which aimed to encourage patients with the provision of information about the importance of mobilising as well as advice about what kind of activities they could do while in hospital. An online learning resource was developed for nursing and HCA staff which sought to incorporate the philosophy of End PJ Paralysis into the culture of care on the ward. This was complimented by posters displayed throughout the ward with prompts and tips encouraging patients and staff to engage. Implementation is ongoing and initial PDSA evaluation of audit data from the IR initiative is demonstrating improvement in mobility outcomes. There has been a notable increase in the number of patients sitting out of bed and dressed on the ward as well as the numbers of patients mobilising.

### Access to food and hydration

The co-design team noted that the risk of malnutrition while in hospital increases with the degree of frailty of the patient and is associated with reduced staffing resources as well as significant food wastage in the hospital site. The PPR members of the co-design team reflected on their experiences of hospital care where water cups were placed out of reach and where lack of assistance with feeding meant that food trays were collected by staff untouched. They noted the important role that family carers often provide with assisting a frail patient with eating however remarked that visiting hour structures may act as a barrier. They observed that access to food and water should be a basic element of quality care.

It was agreed by the team that a series of interventions along the pathway of acute care for an older person would be required to address this service delivery area. The team discussed structural issues such as placement and refilling of water jugs and cups, the use of bedrails on emergency department trolleys as well as the availability of nursing and HCA staff to assist with meal times as barriers to adequate nutrition and hydration among frail older people. It was agreed by the team that signs about visiting hours within wards should clearly state that family carers who wish to assist their relative at meal times are welcome and should speak to a ward nurse to arrange this.

The co-design team observed that a dedicated HCA role was required in order to improve patient access to hydration in the Emergency Department. This role should be incorporated into the FITT early intervention implementation. In response to this suggestion by the co-design team, two HCA roles within the ED have been assigned to address the needs of frail older patients (hydration and toileting). Furthermore, it is intended to provide cup holders as an attachment to the trolleys in the ED which would allow for the placement of water cups within reach of patients.

Access to water was also embedded into the IR programme whereby nurses were asked to assess during rounding whether patients could reach their water and was the jug filled. The catering department have introduced a new design of water jug which is light weight and therefore easier to lift. Furthermore, this jug is transparent which facilitates monitoring of water volume and prompt refilling.

A Red Tray intervention was piloted in a geriatric ward within the hospital with the aim of improving the nutrition and hydration of patients. This involves significant cross disciplinary case management including catering, dietetics department, nursing and HCAs. Patients who require assistance at meal times should be identified by nursing staff on the ward and this should be communicated to the catering team. These patients’ food is then delivered to the ward on a red tray to distinguish it from the other patients’ food. This should trigger the catering staff to hold back food on a red tray until a nurse or HCA is available to provide assistance to the patient. The success of the pilot intervention was demonstrated through PDSA audit data which observed improved protein and energy consumption among frail older patients following introduction of the Red Tray. There is a plan for a phased hospital implementation of the intervention beginning with extension to all geriatric wards, then to single room wards, the AMU and subsequently hospital wide.

### Improved patient information and hospital signage

Communication of information from healthcare professionals to patients or family carers was highlighted by the co-design group as a critical area for improved service delivery for frail older people. The PPR members of the co-design team provided powerful accounts detailing instances where poor communication from healthcare teams resulted in negative patient experiences of care, including safety risks. This was stressed as being particularly challenging for a person with a cognitive impairment. The PPRs framed these accounts in terms of patient dignity and respect. They acknowledged that healthcare staff in acute settings are often experiencing high levels of stress and, in some instances burnout, due to resource constraints and a burdened health system. However, communication of information to patients was identified as a priority area by the group as it was critical to the patient’s overall experience of acute care as well as patient safety.

The PPR members suggested that written daily care plans should be incorporated into the patient notes and remain with the patient after consultant morning rounds. This would allow the patients to review the physician recommendations and prescriptions throughout the day. In response, consideration is being given to adapting the ‘what matters to you’ initiative. This is a nursing quality improvement initiative developed for implementation within nursing education and practice development [32]. It was agreed that rather than healthcare staff being asked to document elements of a daily care plan, patients and/or their family carers could be empowered to document what is important to them on a self-complete card which could remain at their bedside. This would facilitate more person-centred care for patients, particularly for patients who are experiencing delirium or cognitive impairment during their admission. The cards may aid communication about what is important for patients and assist healthcare teams to adopt a person-centred approach to their communication. The card would also include a comments section in which healthcare professionals could write notes to the patient which would prompt mobility prescribing for that day and/or clarify elements of the patient's diagnosis, prescriptions or diagnostic tests.

Under the priority area of improved patient information, it was agreed that patient information dissemination material should be co-designed with PPRs in order to assist patients in preparing for their hospital admission particularly in relation to mobility, hydration and nutrition. The group also noted the difficulty in negotiating complex hospital environment and questioned whether an intervention could be developed which would assist patients to find their way around the building more easily. The co-design team are currently liaising with the hospital Environmental Dementia Committee in order to develop and undertake an audit evaluating the hospital signage and communication from the perspective of frailty and cognition.

### Summary of findings

The quality improvement initiatives which are being delivered within the SAFE pathways correspond to five specific co-designed priority areas for service improvement for frail older people. The co-design discussions prompted the introduction of patient-centred quality improvement initiatives which targeted cultures of care, social relationships as well as environmental structures in the acute setting.

The targeted interventions for service and quality improvement which sought to enhance a culture of personalisation in the care of older people included improved inter-professional collaboration with early and precise identification of frail patients and a common understanding of frailty. Furthermore, the co-design team called for improved information sharing across relevant disciplines of care as well as across organisational boundaries of care (community to acute). In addition, person-centred communication of information to patients and their families was identified for improvement initiatives.

The service and quality improvements initiatives which were prompted by the co-design discussions targeted environmental and social restructuring of care delivery to frail older people. This included improved hospital signage and the development of co-designed patient information dissemination material as well as self-complete information cards to assist with care plan documentation. The study also identified further structural issues for intervention such as; the necessity to evaluate the placement of cups within reach, the barrier to access hydration posed by guard rails on trolleys and the need to allocate resources to facilitate assistance at meal times in order to reduce food waste and target patient morbidity through improved nutrition. Interventions such as IR, FITT, the Red Tray and End PJ Paralysis resulted in a social restructuring of dedicated roles within frontline nursing and allied health staff to the priority areas of mobility, continence, nutrition and hydration. This restructuring and alignment with patient-centred outcomes requires adequate resourcing (including staffing) and the commitment of organisational leaders.

## Discussion

A key strength of the quality improvement initiatives embedded into the SAFE pathways for frail older people is the co-design approach which places patient centred outcomes such as dignity, identity as well as independence as key drivers for implementation [15, 23] . Nelson et al. [33] have noted the dearth of evidence demonstrating how patient reported outcomes can be used to improve quality of care from the patient’s perspective. Each of the initiatives implemented within the SAFE pathways were aligned to co-designed priorities and patient-centred improvement outcomes. Measurement and evaluation of these outcomes, while challenging, should be core to the phased implementation of these initiatives in the hospital site and the evaluation of the quality of care for frail older people in acute settings.

The cultural and behavioural factors influencing communication between patients, family carers and healthcare professionals was identified in the SAFE study as being of critical importance to improving service delivery for frail older people. Effective communication and shared decision-making are core concepts of integrated person-centred care [34, 35]. Research evidence has demonstrated a connection between improved patient safety and quality outcomes and a culture of healthcare which is characterised by collective leadership and strong interpersonal relationships across healthcare professionals and disciplines [35, 36].

Audit data produced during the PDSA cycles evaluating the impact of both the FITT intervention and the IR initiative identified improved patient outcomes in relation to early intervention, length of stay and mobility. The identification of IR champions (one staff nurse, one HCA) charged with the role of promoting the initiative among ward colleagues and with demonstrating the IR process was shown to be critical for the adoption of IR into nursing practice at the pilot site. Challenges to the sustainability of this intervention as it rolls out across the hospital have been offset through the implementation evaluation phase. In particular, the development of educational materials including a video, an online learning module, the installing of poster prompt boards and the fostering of IR champions across the hospital have been key to sustainability [30]. Sustainable phased implementation across the hospital for both interventions are planned for 2019.

The SAFE study targeted cultures of care for frail older people through co-designed initiatives which sought to improve the personalisation of care through better communication of information with patients as well across disciplines and organisational boundaries. Identification and measurement of outcomes aligned to cultures of care is challenging. These outcomes should include improved coherence along the care journey through information sharing, patient handover protocols and inter-disciplinary case management [35]. The embedding of person-centredness into every interaction between a patient and the health system along their care pathway is central to this cultural transformation.

### Reflections

Many of the improvement initiatives introduced in the SAFE study involve cross disciplinary collaborative working between multiple healthcare professionals. This necessitates a negotiation of roles and responsibilities as well as collective leadership across these professional areas [21, 36]. Education and training are critical to encouraging professional commitment to the initiatives as well as ongoing collaboration. Interdisciplinary education and training with regard to the collective case management approach to managing frailty along a care continuum is an important priority area for future intervention [14, 36]. Key to the success and future sustainability of the many of the SAFE initiatives is the identification of a staff champion who can be a liaison between the different professions while negotiating disciplinary responsibilities and demonstrating strong interpersonal communication skills.

Improved access to hydration and nutrition as well as a commitment to better continence care necessitate interventions which target the culture of care for frail older people in acute hospital settings [14, 15, 30]. Continence care is fundamental to the nursing role and a central domain of competence for nursing practice development. It is an aspect of quality nursing care that may be compromised in the context of staff shortages and scarce resources [11]. In the SAFE study successful implementation of practice change in the areas of continence care, improved mobility as well as access to food and hydration required facilitation by key organisational leadership and collaboration. In particular, this study involved the strategic oversight of nursing leadership within the hospital in order to embed Intentional Rounding (IR) into nursing practice development.

The PPR voice in the co-design process for the SAFE project was found to be critical in focusing attention of the organisational leadership on the priority areas that were identifed by the PPRs. International evidence has noted that the inclusion of organisational leadership in co-design and implementation teams is a critical factor in the success of interventions targeting service delivery and quality improvement [21, 22, 28]. This was particularly important for opening channels of communication and improving relationships, trust and empowering of personnel involved in the case management of frail older people along the care continuum from primary to tertiary care.

Adequate resourcing and policy administrative commitment pose the greatest challenges to the sustainability of the interventions developed along the SAFE pathways. Investments in the fostering of inter professional collaboration, role recognition and knowledge exchange between healthcare professionals requires ongoing commitment from organisational leaders and management [34]. Challenges to the implementation of FITT may arise from resource issues pertaining to staffing particularly as therapists are drawn away from the ward teams to the emergency department. End PJ Paralysis, as an element of the IR intervention, requires a shift in the culture of hospital care and will necessitate education for both patients and healthcare staff in relation to the benefits of getting dressed and out of bed while in hospital. Strategic organisational leadership and commitment is therefore critical to successful implementation of both initiatives [27]. PDSA audit data demonstrating benefits in terms of improved patient-centred outcomes is critical.

### Strengths and limitations

A major strength of this study was that, via the co-design process, the participants noted that they felt they could make an important contribution to an area of health service delivery which they felt strongly about and were directly affected by. Furthermore, the clinical participants were open to viewing the health system through the lens of the patient or public and noted the importance of placing the patient experiences at the heart of service delivery. This was particularly evident when it came to prioritising service areas for intervention along the pathway. Communication in particular was identified as a key priority area by the co-design team and much of the resulting intervention areas target more effective, respectful and clear communication between healthcare personnel and patients. This was an unanticipated target area for the pathway that was user driven from the perspective of the co-design team and was an area for very fruitful discussion and co-learning.

The inclusion of organisational leadership in the co-design and implementation teams was a critical factor in the success of the interventions targeting service delivery and quality improvement. This was a key strength for this study and should be a significant feature of any co-designed service or quality improvement effort.

The identification of public and patient representatives for the co-design team was facilitated through third party community or voluntary sector organisations. While this ensured a diverse range of experience was represented in the co-design group it may also represent a limitation for this study. It is possible that the co-design team may be overly representative of public or patients who are engaged and interested in quality improvement and who have the cognitive and socio-economic capacity to engage with this type of co-design work.

The limitations of the co-design approach include its complex non-linear design. This necessitates the consideration of local contexts before adoption of the design approach [21]. This may limit the transferability of the study findings beyond the acute care setting as well as to other patient population groups. The co-design method requires considerable commitment from all involved in terms of time and resources. For the process to be transferable there has to be a shared understanding from the outset that outcome measures and timeframes cannot be pre-specified in a co-design process [2, 21].

## Conclusion

The aim of the project was to co-design with public and patient representatives (PPRs) enhanced integrated care processes and service delivery for frail older patients in an acute tertiary hospital. This was conceptualised as acute care pathways for older people which integrate community based primary care referral processes as well as secondary and primary care discharge pathways. Using a co-design approach we facilitated a process of democratic dialogue in the development of quality improvement initiatives which were responsive to patient-centred outcomes. Co-learning and recognition of mutual benefit were at the core of the co-design process.

## Data Availability

The datasets (transcribed recordings of co-design meetings) presented in this publication are available from the corresponding author on reasonable request.

## Appendix 1

The first four co-design meetings for the SAFE study consisted of:

1. Introductions, capacity building (project familiarity) and agreement of terms of reference for co-design participant roles and objectives. The co-design team were provided with a descriptive summary of the current service delivery pathways for older people through the relevant hospital site.
2. In-depth discussion of data focussed on PPR experiences and understandings of frailty in later life as well as health service delivery for older people in the Irish context
3. Consolidation and prioritisation of co-design team recommendations and adaptations to the proposed model care pathway
4. Identification of patient-centred outcomes for pathway adaptation and evaluation

The final two workshops were conducted alongside the pathway evaluation through a series of intervention Plan-Do-Study-Action (PDSA) cycles (XXXX). The process involved the gathering of evaluative testing data relevant to the patient-centred outcomes identified by the co-design team. The final two co-design meetings were structured as follows:

5. Assessment of patient-centred outcomes and reporting of the PDSA data in relation to pathway implementation and enhanced service delivery
6. Review of the co-design process and development of recommendations for the sustainable ongoing implementation of the pathway within the local site and beyond. Identification of dissemination preferences targeting public and patients as well as the wider health system.

## Appendix 2

**Table 2:** Discussion extracts related to five priority service improvement areas

Priority Area	Key PPR Quotes
Collaboration along an integrated care continuum for frail older patients	“Often there is a reluctance of people to be discharged unless there is a safety net. People want to leave hospital but often there isn’t a support structure and it can all break down again.” “We have talked about other hospitals not communicating and sharing databases but even with the A&E from reception to the [assessment area] to be assessed again you have to provide all your details again.”
Continence care	“Do they need to be on a trolley? Because the trolley means the sides are up and the person is immediately dis-empowered.” “Can you get yourself to the bathroom? You are on a trolley and a nurse can’t come to you every time and all of a sudden it’s a serious thing.” “I’m still horrified by the idea that I’m but up on a trolley as a default. I’ve come in with something and all of a sudden … the critical thing for me is if I can get to the loo or not.... And if I can’t I’m put in this situation that I’m in a … cot with bars that I haven’t been in since I was 18 months. I can’t go to the bathroom and I turn into an infant in the space of a couple of hours. I think we need to take a chance on people because the outcome can be catastrophic on the person.”
Improved mobility	“It’s a huge amount to do with self-esteem. The difference between seeing a visitor when you’re in your pyjamas and seeing a visitor when you’re in your choice of clothes […] we all want to appear well.” “If I’m well enough [to be out of bed then] lying on my bed in my pyjamas is not conducive in getting home quickly.”
Access to food and hydration	“I’d think catering staff would have found it really hard [before bringing them on board], to be taking in [patients’ meals] and not actually be allowed to assist somebody.” “Access to water is important as dehydration amongst frail patients can lead to critical conditions like delirium.” “Important that these changes do not fade with new staff. Learning units and inductions are mandatory for new staff, so having these changes integrated.”
Improved patient information and signage	“You are so vulnerable when you come into hospital … and a lot of it can be improved with just communication.” “Communication is key. A whole lot of anxiety can build up. They had a test on Monday and its now Wednesday and someone says “oh we will get back to you” but people are anxious by virtue of being in hospital even with its not very serious. They need to know all the time of what the stage of investigation is.”

## Abbreviations

ED: Emergency Department
FITT: Frailty Intervention Therapy Team
HCA: Healthcare Assistant
ICT: Integrated Community Care Team
IR: Intentional Rounding
PDSA: Plan, Do, Study, Act
PPI: Public and Patient Involvement
PPR: Public and Patient Representative
SAFE: Systematic Approach to improving care for Frail older patients
